# Genome-Wide mRNA and lncRNA Expression Profiling to Uncover Their Role in the Molecular Pathogenesis of Developmental Dysplasia of the Hip

**DOI:** 10.3390/ijms26168058

**Published:** 2025-08-20

**Authors:** İbrahim Kaya, Mine Türktaş, Semih Yaş, Resul Bircan

**Affiliations:** 1 Dr. Abdurrahman Yurtaslan Ankara Oncology Training and Research Hospital, Department of Orthopaedics and Traumatology, Demetevler, Vatan Cd., Yenimahalle, Ankara 06200, Türkiye; drsemihyas@gmail.com (S.Y.); resul_bircan@hotmail.com (R.B.); 2 Department of Biology, Faculty of Science, Gazi University, Ankara 06560, Türkiye; mineturktas@gmail.com

**Keywords:** developmental dysplasia of the hip, long non-coding RNAs (lncRNAs), transcriptomic analysis, molecular pathogenesis, epigenetic factors

## Abstract

Developmental dysplasia of the hip (DDH) is a congenital disorder influenced by genetic and epigenetic factors. This study aimed to elucidate the molecular pathogenesis of DDH through a comprehensive transcriptomic analysis, identifying differentially expressed genes (DEGs) and long non-coding RNAs (lncRNAs) in hip joint capsules from DDH patients and healthy controls. RNA sequencing data from 12 samples (6 DDH, 6 controls) were retrieved from the NCBI database. Functional annotation was performed using Gene Ontology (GO) and Kyoto Encyclopedia of Genes and Genomes (KEGG) pathway analyses via the DAVID tool. A protein–protein interaction (PPI) network of DEGs was constructed using STRING with medium confidence settings. Among 78,930 transcripts, 4.3% were significantly differentially expressed, according to DESeq2 analysis. A total of 3425 DEGs were identified (FDR < 0.05, |log2 FC| > 2), including 1008 upregulated and 2417 downregulated transcripts in DDH samples. Additionally, 1656 lncRNAs were detected among the DEGs. These findings enhance our understanding of the genetic and epigenetic landscape of DDH and highlight the involvement of key biological pathways such as cell cycle regulation and Wnt signaling. This study provides a foundation for future molecular research into the pathogenesis of DDH.

## 1. Introduction

DDH refers to a spectrum of abnormalities affecting the hip joint, ranging from mild acetabular dysplasia to subluxation and complete dislocation of the hip [1]. The incidence of DDH varies significantly depending on geographic region and ethnic groups, ranging from 0.06 to 76.1 per 1000 live births [2]. DDH has a complex etiology, and its pathogenesis is not yet fully understood. It is considered a multifactorial disorder involving a combination of genetic, environmental, and mechanical factors [3]. Several risk factors have been identified for DDH, including breech presentation, female sex, first-born status, positive family history, high birth weight, foot deformities, multiple gestation, and oligohydramnios [2,3].

Despite ongoing research into the genetic pathogenesis of developmental dysplasia of the hip, the precise genetic mechanisms underlying the disease remain incompletely understood [3,4,5,6,7]. Several genes have been reported to show a strong correlation with DDH, including *GDF5*, *HOXB9*, *HOXD9*, *PAPPA2*, *COL1A1*, *ASPN*, *CX3CR1*, *UQCC*, *POLE*, *TBX4*, *MMP24*, and *NOTCH2* [3,4,5,8,9]. Although these genetic variations have been found to be associated with DDH, their exact impact on disease development remains unclear, and no gene has been definitively identified as being causally linked to the pathogenesis of DDH [3,4,6]. Due to the genetic uncertainty surrounding DDH, gene expression analyses have been conducted to gain new insights into the underlying molecular mechanisms. These studies have identified numerous genes that are differentially expressed and potentially involved in the pathogenesis of DDH [10]. Recently, the role of epigenetic mechanisms—known to be involved in various biological processes—has also been investigated. Findings have shown that both transcriptional and epigenetic regulations may serve as potential modulatory mechanisms in DDH, providing new clues toward understanding its molecular basis [7,11]. On the other hand, there is still a meager number of studies in this field, both at the genome-wide gene expression level and from an epigenetic perspective. A review of the literature shows that, to the best of our knowledge, only a single transcriptome-level (RNA-seq) study has been conducted on *Homo sapiens* to investigate the pathophysiology of DDH [10].

With the advancement of next-generation sequencing technologies, the discovery of various non-coding RNA types, such as lncRNA, has marked significant progress in the field of molecular biology. LncRNAs, a class of non-coding RNAs with more than 200 nucleotides, do not have protein-coding potential but play crucial roles in maintaining cellular homeostasis. They are involved in regulating gene expression at the epigenetic, transcriptional, and post-transcriptional levels, and play key roles in various biological processes such as somatic cell reprogramming and the pluripotency of stem cells [12,13]. Recent studies have shown that there are more than 100,000 distinct lncRNAs encoded by the human genome [14,15]. The expression of lncRNAs has been associated with a range of diseases, including cancer and neurological disorders, highlighting their significance as key factors in both normal and pathological conditions [12,13,15,16]. In recent years, evidence has emerged suggesting that lncRNAs play a crucial role in the proliferation and differentiation of osteoblasts and chondrocytes [17,18,19].

LncRNAs have been shown to regulate gene expression, playing a significant role in osteogenic differentiation and bone regeneration. In particular, LncRNA H19 has been found to play a vital role in osteogenic induction [19]. As the effects of lncRNAs on the skeletal system have become more evident, studies investigating the relationship between DDH and lncRNAs at the genomic level have emerged. These studies have begun to reveal promising data regarding the epigenetic mechanisms underlying DDH [20,21].

To the best of our knowledge, this study is the first to investigate the molecular pathogenesis of DDH by using genome-wide transcript and lncRNA profiling. In this study, transcriptomic libraries available in the database were analyzed using various bioinformatics tools to identify transcripts and lncRNAs as epigenetic regulators which are involved in the pathogenesis of DDH. Both transcripts and lncRNAs were identified, and their potential roles in DDH development were discussed.

## 2. Results

### 2.1. Mapping Efficiency

To ensure the reliability of the in silico RNA-seq analysis, standard quality control thresholds were applied. Raw reads were subjected to adapter trimming and low-quality base removal, and FastQC reports confirmed high per-base sequence quality (Q ≥ 30), low adapter contamination, and appropriate GC content. Alignment to the Ensemble reference human genome GRCh38 was performed using HISAT2, with an average overall mapping rate exceeding 88%. The proportion of reads that mapped to the Ensemble reference genes ranged from 81 to 98% for the 12 samples (Table 1).

### 2.2. Differentially Expressed Genes in Hip Joint Capsules of DDH Patients

After mapping, 78,930 transcripts were identified. The read counts of 78,930 transcripts were calculated to normalize the expression level of the transcripts. Using DESeq2 with a paired analysis design, 4.3% of all genes were identified as significantly differentially expressed, demonstrating a substantial number of gene expression differences between the DDH patients and control groups. A total of 3425 DEGs were identified between DDH vs. the control samples based on the selection criteria (a false discovery rate < 0.05 and |log_2_ FC| > 2) (Table S1).

**Figure 1:**
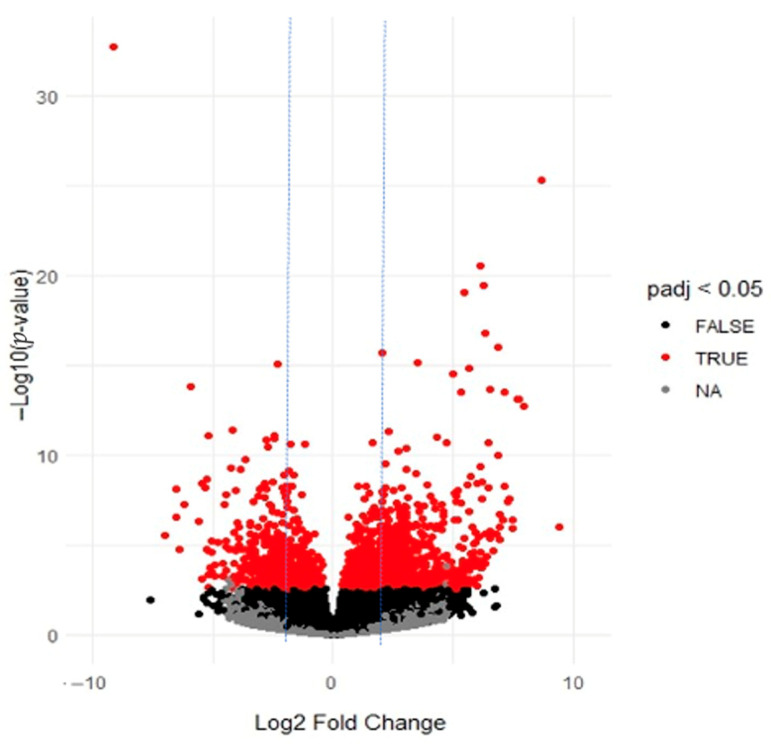
Distribution of transcripts shown as a volcano plot. Each point represents a gene. The red dots represent the differentially expressed genes (cutoff: |log2FC| > 2 (blue line), padj < 0.05).

The differential expression analysis of the transcripts between the samples revealed that most of the transcripts exhibited downregulation in DDH patients compared with the controls. It was found that 1008 transcripts were upregulated, while 2417 transcripts were downregulated in DDH patients. Figure 1 presents a volcano plot to examine the difference in the expression level of genes in two groups of samples and the statistical significance of the differences. Moreover, the top 50 up- and downregulated genes are listed in Table 2.

**Figure 2:**
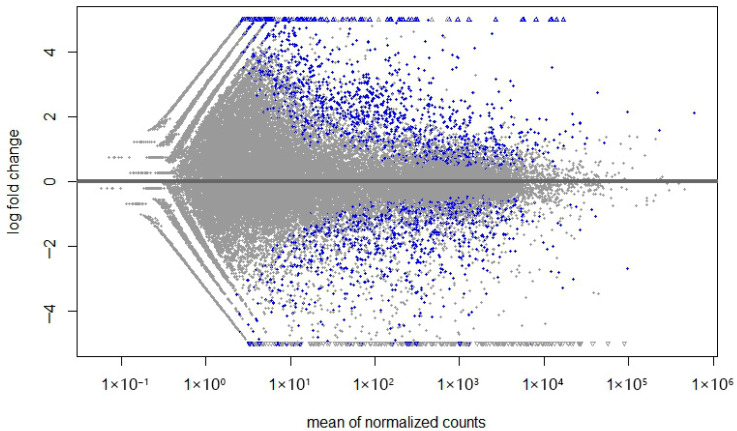
PA plot from the transcripts. Each transcript is represented by a dot. The *x*-axis is the average expression over all samples, and the *y*-axis is the log2 fold change between the DDH and control groups.

The plotMA function of DESeq2 was applied to obtain the log2 attribution to the given variables over the mean of normalized counts for an experiment with a two-group comparison (Figure 2).

### 2.3. Functional Enrichment Analysis of DEGs

**Figure 3:**
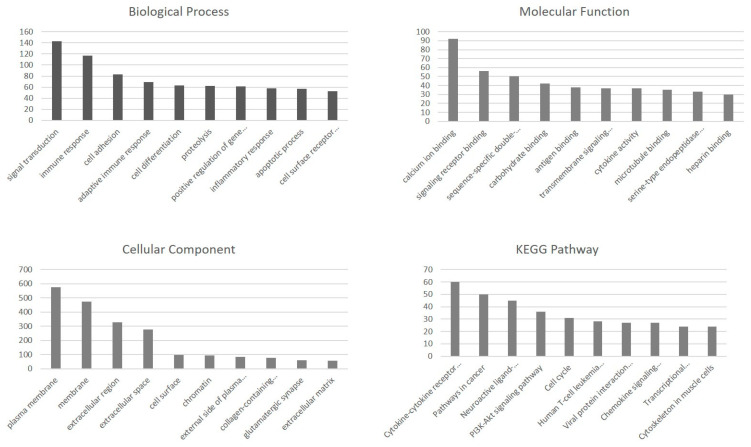
Top 10 enriched BP/MF/CC GO terms and KEGG pathways of DEGs between the DDH and control samples.

DEGs identified between the DDH and control samples were analyzed using GO term enrichment to investigate their functions (Figure 3). The transcripts were classified on the basis of molecular function (MF), biological process (BP), and cellular component (CC) ontology terms.

A list of 345 biological process GO terms and 41 pathways were retrieved. DEGs are mainly involved in signal transduction following immune response and cell adhesion. Several transcripts were assigned to GO CC terms, and the top three CC terms are plasma membrane, membrane, and extracellular region. The most enriched MF term is calcium ion binding.

Pathway enrichment analysis using the KEGG database was also performed to reveal the active biological pathways involved in DDH patients compared with healthy individuals. KEGG pathway analysis revealed that the cytokine–cytokine receptor interaction pathway is the most abundant pathway.

**Figure 4:**
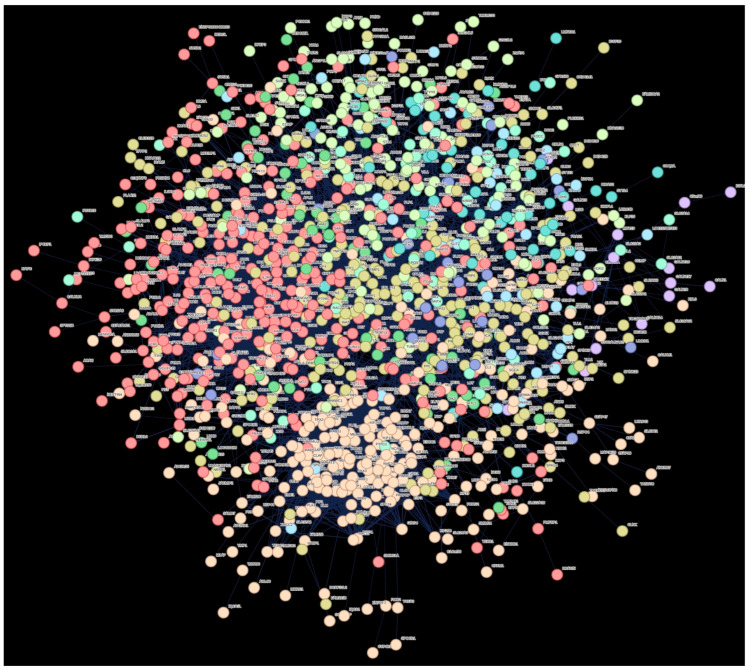
Protein–protein interaction network analysis. The transcripts are grouped into 10 clusters. Each color represents one group.

DEGs were also subjected to STRING analysis to investigate the PPI. K-means clustering was applied, and the top network in the control and DDH groups are presented in Figure 4.

Cell communication was found to be one of the top 10 most common biological processes, while the cytokine–cytokine receptor interaction pathway was found to be one of the most significant pathways. The descriptions of the clusters are listed in Table 3.

### 2.4. Identification of Long Non-Coding RNAs

Since the long-noncoding RNAs have various functions, in order to analyze the contribution of m-lncRNAs to DDH, they were also identified in the study. A total of 1656 lncRNAs were determined among the DEGs (Table S2). Moreover, the top 100 up- and downregulated lncRNAs are listed in Table 4. The presence of a large number of lncRNAs among the DEGs demonstrated that they have important roles in DDH disease.

## 3. Discussion

In this study, bioinformatic analyses were conducted to elucidate the genetic and epigenetic mechanisms of DDH. One of the most striking findings of the study was the identification of 3425 differentially expressed genes in DDH, with 1008 being upregulated and 2417 downregulated. Additionally, Gene Ontology and KEGG pathway analyses revealed that the regulation of various signaling pathways is implicated in the molecular pathogenesis of DDH. Another significant finding was the observation of significant expression changes in 1656 lncRNAs in DDH, with 433 lncRNAs showing increased expression and 1223 lncRNAs showing decreased expression.

In the present study, it was found that the expression of *Matrix Metalloproteinase13* (*MMP13*) and *Matrix Metalloproteinase3* (*MMP3*) was reduced in the hip joint capsule of patients with DDH. Similarly, a transcriptomic study conducted in 2021 reported decreased expression of *MMP1*, *MMP3*, *MMP9*, and *MMP13* in the joint capsule of DDH patients, which is consistent with the findings of our study [10]. However, these results are intriguingly different from studies showing increased expression of *MMP13* and *MMP3* in the cartilage of DDH patients [21,22]. In a study investigating biomarkers of cartilage degeneration in DDH, an increase in *MMP13* expression in the joint cartilage matrix of DDH patients was found, and it was revealed that cartilage degeneration progresses in these patients [23]. The effects of *MMP13* and *MMP3*, members of the matrix metalloproteinase family, on cartilage degeneration are also supported by other studies [21,22]. A study on human osteoarthritic cartilage reported that *MMP* expression in chondrocytes varies according to the depth of chondrocytes in the cartilage and the severity of the disease [24]. Histological differences between capsule and cartilage tissues suggest that *MMP* expression may play tissue-specific roles. Additionally, as shown in osteoarthritis patients, the stage of DDH may also influence *MMP* expression. This situation may be the main reason for the conflicting results regarding *MMP* expression between capsule and cartilage tissues reported in the literature.

The *Growth Differentiation Factor 5* (*GDF5*) gene has been identified as an important candidate gene associated with DDH in numerous studies [3,8,9,25]. It has been shown that *GDF5* plays a critical role in joint and bone formation and supports the condensation of mesenchymal cells [8,25]. Reduced *GDF5* expression has been suggested to impair the condensation of these cells and chondrogenic differentiation, potentially leading to a decrease in the number of chondrogenic cells in the hip joint [25]. A study investigating the effects of *GDF5* stimulation on chondrocytes demonstrated that *GDF5* stimulation had an inhibitory effect on the expression of catabolic genes and a stimulatory effect on the expression of anabolic genes in human articular chondrocytes. It was shown that *GDF5* stimulation inhibits the canonical Wnt signaling pathway via *DKK1* (Dickkopf WNT signaling pathway inhibitor 1), thereby reducing Wnt-induced *MMP13* expression and preventing its catabolic effects on chondrocytes. Furthermore, when *DKK1* was inhibited, there was a subsequent increase in *MMP13* expression, which was associated with an increase in chondrocyte apoptosis [26]. Conversely, some studies have reported that *DKK1* stimulation increases chondrocyte apoptosis and cartilage degradation [27,28]. In conclusion, all these findings suggest that *DKK1* plays a regulatory role in chondrocyte homeostasis.

The *DKK1* gene has been identified as a candidate gene associated with DDH in several studies [3,4,8]. In the present study, it is noteworthy that the expression of an lncRNA (lncRNA activating regulator of *DKK1*), which has a regulatory effect on the *DKK1* gene, was found to be reduced. This finding supports our hypothesis that lncRNAs may play a role in the epigenetic mechanisms of DDH. The *Wnt Inhibitor Factor 1* (*WIF1*) gene inhibits the WNT signaling pathway, which plays a crucial role in embryonic bone and joint development. The relationship between this gene and DDH was first demonstrated in a study conducted in the Chinese population in 2019. According to the results of that study, it was found that the expression of the *WIF1* gene was significantly increased in the joint capsule and ligaments of DDH patients [29]. In our study, we also found that the expression of the *WIF1* gene was increased in the DDH joint capsule. In this context, our study confirms the relationship between *WIF1* and DDH. The study conducted in the Chinese population suggested that the excessive expression of *WIF1* in DDH patients could be associated with the reshaping of the hip’s macro-morphology through the suppression of Wnt signaling [29]. In contrast to previous studies, our research found that genes regulating the inhibition of Wnt signaling pathways, or lncRNAs that regulate these genes, were affected in the opposite direction. While the increase in *WIF1* gene expression contributes to the inhibition of Wnt signaling, the decreased expression of “lncRNA activating regulator of *DKK1*”, the transcriptional activator of *DKK1*, may lead to a reduction in *DKK1* levels and consequently a decrease in the inhibition of the Wnt signaling pathway. These findings suggest that both excessive activation and inhibition of the Wnt signaling pathway may play a role in the development of DDH. More comprehensive functional studies are needed to fully understand the impact of the Wnt signaling pathway on DDH’s pathogenesis.

It is well known that Wnt signaling cascades play a role in the development of cartilage, bone, muscle, and joints [30]. Although alterations in Wnt signaling have been reported to adversely affect chondrocyte differentiation and endochondral ossification, its exact role in joint development remains unclear. There are contradictions among reports in the literature, as Wnt’s ligand expression, target cells, and signaling pathways are complex [31]. In our study, the conclusion that Wnt signaling pathways may be affected differently can be explained by the fact that these pathways are in complex interactions with numerous regulators. Additionally, it has been shown that the Wnt signaling pathway is one of the affected candidate pathways in DDH [7]. The fact that genes with regulatory effects on the Wnt signaling pathway, such as *DKK1, FRZB,* and *WISP3*, have also been identified as candidate genes for DDH further supports the potential role of this signaling pathway in the pathogenesis of DDH [8].

The Wnt signaling pathway is negatively regulated by various soluble factors, such as *WIF1* and *DKK*, in the extracellular environment [29]. In our study, it was found that both of these genes, which are influential in the Wnt signaling pathway, were affected in patients with DDH. Additionally, the results of the GO analysis revealed that the most affected biological process was signal transduction, the molecular functions were calcium ion binding and signaling receptor binding, and among the top 10 affected cellular components, the extracellular region, extracellular space, and extracellular matrix were identified. These findings suggest that extracellular matrix-dependent signaling mechanisms and the extracellular microenvironment may play important biological roles in the pathogenesis of DDH. In the literature, calcium ion binding is well known to play a critical role in both bone remodeling and growth, as well as in intracellular signal transduction. Ca^2+^ is involved in a wide range of cellular processes such as mitosis, neuronal transmission, gene transcription, and cell death, and it plays a particularly important role in the regulation of gene expression. In osteoblasts, calcium-mediated signaling pathways are among the key mechanisms regulating cell proliferation and differentiation [32]. Similarly, Ca^2+^ signaling in osteoclasts is essential for various cellular functions, including differentiation, bone resorption, and gene transcription. Recent studies have highlighted the importance of intracellular Ca^2+^ signaling for osteoclast differentiation and shown that this process initiates osteoclast-specific gene transcription to promote differentiation [33]. Additionally, in cells of the osteoblastic lineage, Ca^2+^ signals, taken up via calcium channels in response to both mechanical and hormonal stimuli, affect gene expression and cellular behavior, thereby contributing to bone homeostasis. Calcium signaling, which regulates the interaction between osteoblasts and osteoclasts, also plays a critical role in the modulation of bone remodeling. These signals are associated with the activation of intracellular signaling pathways that control cell behavior and phenotype, including gene expression patterns [34]. In this context, the finding that “calcium ion binding” was among the most affected molecular functions in our study of DDH cases suggests that calcium signaling and/or calcium-dependent cellular processes may be impaired in this disease.

Cytokines and cytokine receptors are important proteins involved in a wide range of physiological processes such as mediating cellular communication, initiating and regulating inflammation, and controlling cell proliferation, differentiation, and apoptosis. The effects of cytokines such as IL-1β, IL-6, TNF-α, and TGF-β on the skeletal system are known [7,10,19,35]. For example, TGF-β signaling has been shown to play an important role in embryonic skeletal development as well as postnatal bone and cartilage homeostasis [35]. Additionally, in the hip joint capsule of patients with DDH, TGF-β1 has been reported to be downregulated while TGF-β2 is upregulated [10]. In our study, one of the most enriched pathways in the KEGG analysis was the “cytokine–cytokine receptor interaction” pathway. These findings suggest that alterations in cytokine signaling pathways may play a role in the pathogenesis of DDH.

Previous studies have reported that *Myocilin* (*MYOC*), a gene known to be associated with glaucoma, functions as a modulator of the Wnt signaling pathway [36,37]. In the present study, an increase in the expression of 1,008 transcripts was observed, with *MYOC* being the gene showing the most significant increase. Given the modulatory effect of *MYOC* on the Wnt signaling pathway, it is likely to be associated with the pathogenesis of DDH. This is the first study to demonstrate a relationship between the *MYOC* gene and DDH.

*Iroquois Homeobox* (*IRX*) genes belong to the homeobox family and encode homeobox proteins. *Irx* genes play crucial roles in regulating various developmental processes, particularly during embryonic development, where they are involved in cell specification, cell differentiation, and the organization of body structures [38]. Additionally, a study conducted in 2010 demonstrated that *IRX3* is transcriptionally induced by the Wnt signaling pathway during neurulation [39]. In this study, the expression of the *IRX1* gene was found to be the fifth most increased among the genes in DDH patients. The significantly increased expression of this gene in DDH patients suggests that it may play a critical role during the embryonic development of the hip joint and could potentially be a direct or indirect regulatory gene in the pathogenesis of DDH.

It is known that *lncRNA-H19* has an effect on osteogenic and chondrogenic differentiation [19,40]. There are only two studies investigating the role of lncRNAs in the epigenetic mechanisms of DDH pathogenesis. These studies have particularly focused on the effects of *lncRNA-H19* on chondrocytes, showing that *H19* could be an important epigenetic regulatory factor in the development of DDH [20,21]. Recent studies have shown an increasing amount of data indicating that lncRNAs play a significant role in various diseases [13,16]. Based on the hypothesis that a greater number of lncRNAs may be involved in the epigenetic regulatory mechanisms of DDH, our analysis revealed changes in the expression of numerous lncRNAs.

Particularly, the interactions of some of these lncRNAs with target genes are noteworthy. For example, the *AFF3* gene regulates the expression of genes involved in mesoderm and ectoderm development, mesenchymal cell proliferation, cell adhesion, angiogenesis, and cartilage and lens development [41]. In our study, the increased expression of the *lncRNA ENSG00000301580*, which has an antisense effect on *AFF3*, suggests that this gene may play a regulatory role in the pathogenesis of DDH. Another lncRNA, *ENSG00000286411*, which also shows increased expression in DDH, exerts an antisense effect on *Cyclin-dependent kinase* (*CDK*) *14. CDK14*, as a cell cycle-dependent protein kinase, is involved in the canonical Wnt/β-catenin signaling pathway, which directs the cell cycle towards mitosis. It has been reported that the suppression of *CDK14* leads to proliferative and migratory defects in human umbilical cord endothelial and epithelial cells [42]. Similarly, in the hip joint, the proliferation, differentiation, and migration of mesenchymal cells are crucial during development. The increased expression of the *lncRNA ENSG00000286411*, which targets *CDK14* in DDH, may contribute to the disruption of these mechanisms. In a transcriptomic study, it was found that the *CDK1* protein, which is essential for the progression of mitosis, is downregulated in DDH patients [10]. This finding aligns with the results we obtained in our study, where the *lncRNA ENSG00000286411* targeting *CDK14* showed increased expression in DDH. In both studies, it has been demonstrated that the regulation of *CDK* family members, which play a role in the mitotic phase of the cell cycle, is disrupted in association with DDH.

The transcriptomic data used in our study were obtained from a publicly available dataset (SRX10431608 through SRX10431613). The dataset includes six patient and six control samples. While this number is limited, it is a common feature in early-stage or pioneering studies utilizing publicly available transcriptomic data.

It should be acknowledged that the differential expression of the identified genes does not necessarily imply a causal role in the pathology of DDH. While some DEGs may act as upstream drivers contributing to disease initiation or progression, others may represent downstream, secondary alterations arising from pathological processes initiated elsewhere. This distinction is particularly important in complex biological systems, where feedback loops and compensatory mechanisms can obscure the temporal sequence of molecular events. We should emphasize that resolving these relationships will require further functional validation studies, ideally complemented by analyses including time-course experiments, functional perturbation studies, single-cell trajectory analyses, and integration with genetic and clinical data, which may help determine whether the observed differential expression reflects a causal role in the pathology or a secondary consequence of upstream events.

On the other hand, while experimental validation is generally needed to confirm computational findings, it is not an absolute prerequisite for the contribution of in silico studies to the scientific literature. It is important to note that in silico analyses provide a valuable framework, especially in emerging or resource-limited research areas, and yield valuable insights and contribute meaningfully to the scientific literature even without direct experimental validation. Due to logistical and infrastructural constraints, we were unable to perform wet-lab validation in the present study. However, this limitation does not negate the significance of these computational results, which are based on well-established bioinformatic pipelines and stringent statistical criteria. Moreover, given that this study is among the first to explore this specific biological context, our findings serve as an initial resource to guide future experimental work. It is widely recognized that computational predictions must be interpreted with caution, yet they often reveal novel candidates and hypotheses that cannot be uncovered without such analyses.

Functional interpretation of differentially expressed lncRNAs remains challenging due to limited annotation and the lack of experimentally validated target interactions in the current databases. As a result, constructing a comprehensive regulatory network was not feasible within the scope of this study. Future research incorporating experimental validation and expanded lncRNA databases will be essential to elucidate the biological roles of these molecules.

## 4. Materials and Methods

### 4.1. Data Acquisition

The raw Illumina RNA sequencing data of 12 samples were retrieved from the NCBI databank. The data were obtained from six hip joint capsules of patients with DDH (SRX10431608–SRX10431613) and six hip joint capsules of control subjects (SRX10431614–SRX10431619).

### 4.2. Transcript Data Analysis

After filtering out low-quality reads (Phred score cutoff: 20) with TrimGalore (https://github.com/FelixKrueger/TrimGalore) (accessed on 2 June 2025), Hisat2 was used to map quality-filtered reads to a reference human genome (hg38) [43]. The aligned read files were further processed by the FeatureCounts program of the SubRead package to obtain read counts [44]. Differential expression analyses were performed using DeSeq2 [45]. DEGs were identified and filtered with the following criteria: false discovery rate < 0.05 and |log_2_ FC| > 2. The Bioconductor BiomaRt R package (version 4.5) was used for lncRNA annotation [46].

### 4.3. Functional Enrichment Analysis

The GO terms and KEGG pathway analysis pathways were discovered as functional annotation categories using the DAVID web-based functional annotation tool [47]. To identify the interactions of DEGs, a PPI network was constructed using STRING (https://string-db.org/) with a “minimum required interaction score” set to medium confidence (0.400). An extensive literature survey was performed to uncover the roles of DEGs in DDH.

## 5. Conclusions

This study is one of the first comprehensive transcriptomic analyses conducted to illuminate the molecular pathogenesis of developmental dysplasia of the hip, identifying various genes with differential expression in the hip joint capsules between healthy controls and DDH patients. The findings from our study suggest that not only protein-coding genes but also lncRNAs may play a significant role in the epigenetic mechanisms underlying DDH’s pathogenesis. Our work provides valuable data that could guide future molecular biological research aimed at understanding the genetic and epigenetic basis of DDH.

## Figures and Tables

**Table 1:** Mapping percentages of the samples.

ID	Treatment	Mapping Rate (%)	Read Number (2 × 150 bp)
SRR14055716	DDH	80.74	24,847,026
SRR14055717	DDH	84.91	33,533,704
SRR14055718	DDH	80.85	23,836,127
SRR14055719	DDH	98.25	27,104,541
SRR14055720	DDH	87.71	24,866,901
SRR14055721	DDH	94.21	30,621,536
SRR14055722	Control	85.35	23,421,990
SRR14055723	Control	93.55	23,402,707
SRR14055724	Control	83.99	25,453,109
SRR14055725	Control	83.48	20,408,199
SRR14055726	Control	91.22	25,169,198
SRR14055727	Control	90.25	21,844,255

**Table 2:** Top 50 upregulated and downregulated differentially expressed genes between DDH and control samples.

ID	log2 Fold Change	*p*-Value	Regulation in DDH
ENSG00000034971	−9.1	1.82 × 10^−33^	Up
ENSG00000309097	−7.6	0.011025698	Up
ENSG00000256513	−7	2.82 × 10^−6^	Up
ENSG00000170549	−6.5	7.46 × 10^−9^	Up
ENSG00000250421	−6.5	2.88 × 10^−7^	Up
ENSG00000279096	−6.4	1.67 × 10^−5^	Up
ENSG00000306524	−6.2	5.87 × 10^−8^	Up
ENSG00000168447	−5.9	1.51 × 10^−14^	Up
ENSG00000240654	−5.6	4.60 × 10^−7^	Up
ENSG00000160097	−5.5	3.54 × 10^−9^	Up
ENSG00000286415	−5.4	0.000715357	Up
ENSG00000305982	−5.4	0.007306598	Up
ENSG00000305491	−5.4	0.010014926	Up
ENSG00000181408	−5.3	2.18 × 10^−9^	Up
ENSG00000179915	−5.3	6.20 × 10^−9^	Up
ENSG00000156076	−5.3	1.73 × 10^−5^	Up
ENSG00000271239	−5.3	0.015421818	Up
ENSG00000142973	−5.2	7.44 × 10^−12^	Up
ENSG00000214866	−5.2	2.19 × 10^−5^	Up
ENSG00000118733	−5.2	0.000432547	Up
ENSG00000274833	−5.2	0.002264803	Up
ENSG00000265962	−5.2	0.004283014	Up
ENSG00000301580	−5.1	5.39 × 10^−6^	Up
ENSG00000188803	−5.1	2.66 × 10^−5^	Up
ENSG00000305239	−5.1	0.000191428	Up
ENSG00000229236	6.5	1.94 × 10^−6^	Down
ENSG00000187689	6.5	4.20 × 10^−6^	Down
ENSG00000149968	6.6	1.96 × 10^−14^	Down
ENSG00000266604	6.7	1.93 × 10^−5^	Down
ENSG00000116147	6.7	0.002750078	Down
ENSG00000211934	6.7	0.025736049	Down
ENSG00000231131	6.8	9.52 × 10^−11^	Down
ENSG00000012817	6.8	2.32 × 10^−6^	Down
ENSG00000211660	6.8	0.023562957	Down
ENSG00000249306	6.9	9.30 × 10^−17^	Down
ENSG00000260197	6.9	1.98 × 10^−7^	Down
ENSG00000229876	7	1.05 × 10^−6^	Down
ENSG00000183878	7	4.61 × 10^−6^	Down
ENSG00000114374	7.1	4.07 × 10^−7^	Down
ENSG00000067048	7.2	2.85 × 10^−14^	Down
ENSG00000126545	7.2	5.33 × 10^−9^	Down
ENSG00000129824	7.3	3.60 × 10^−8^	Down
ENSG00000231535	7.4	2.40 × 10^−8^	Down
ENSG00000211598	7.5	4.07 × 10^−7^	Down
ENSG00000165246	7.5	1.11 × 10^−6^	Down
ENSG00000266995	7.7	6.79 × 10^−14^	Down
ENSG00000291033	7.7	7.11 × 10^−14^	Down
ENSG00000067646	7.9	1.88 × 10^−13^	Down
ENSG00000137745	8.7	5.16 × 10^−26^	Down
ENSG00000198692	9.4	8.86 × 10^−7^	Down

**Table 3:** STRING k-means cluster descriptions.

Cluster Number	Cluster Color	Gene Count	Primary Description
1	Red	339	Immune response
2	Brown	266	Mitotic cell cycle process
3	Dark goldenrod	255	Extracellular matrix
4	Green-yellow	133	-
5	Green 2	65	-
6	Green	56	-
7	Blue	53	-
8	Light sky blue	41	Postsynaptic cell membrane and protein–protein interactions at synapses
9	Medium blue	25	ncRNAs involved in Wnt signaling in hepatocellular carcinoma, and regulation of FZD by ubiquitination
10	Purple	16	O-linked glycosylation of mucins

**Table 4:** Top 100 upregulated and downregulated differentially expressed lncRNAs between DDH and control samples.

ID	BaseMean	Log2 Fold Change	lfcSE	Stat	*p*-Value	padj	Regulation in DDH
ENSG00000309097	20.0553182	−7.6	3.00449956	−2.5418833	0.0110257	0.12197728	Up
ENSG00000256513	12.8646141	−7	1.49363454	−4.6834569	2.82 × 10^−6^	0.00036368	Up
ENSG00000250421	13.1509542	−6.5	1.2755177	−5.1312126	2.88 × 10^−7^	6.37 × 10^−5^	Up
ENSG00000306524	7.35762862	−6.2	1.14212948	−5.4227401	5.87 × 10^−8^	1.76 × 10^−5^	Up
ENSG00000286415	4.37306511	−5.4	1.6074669	−3.3836247	0.00071536	0.02130454	Up
ENSG00000305982	4.31991575	−5.4	2.0210034	−2.6825374	0.0073066	0.09516642	Up
ENSG00000305491	4.18734128	−5.4	2.08974346	−2.5753135	0.01001493	0.1148278	Up
ENSG00000271239	12.0861402	−5.3	2.17536921	−2.4223187	0.01542182	0.1463899	Up
ENSG00000274833	3.70182773	−5.2	1.70130402	−3.0531135	0.0022648	0.04671496	Up
ENSG00000265962	3.6288218	−5.2	1.81060089	−2.8565286	0.00428301	0.07013193	Up
ENSG00000301580	6.83679945	−5.1	1.11596197	−4.5490905	5.39 × 10^−6^	0.00057765	Up
ENSG00000305239	6.90491206	−5.1	1.36677843	−3.7300681	0.00019143	0.00839207	Up
ENSG00000233845	3.34256322	−5.1	1.40746917	−3.5916966	0.00032853	0.01242405	Up
ENSG00000300502	3.29931021	−5	1.71918577	−2.9309225	0.00337957	0.06050065	Up
ENSG00000295404	12.0694117	−5	2.17474927	−2.2856073	0.02227725	0.18085266	Up
ENSG00000307068	4.26292628	−4.9	1.76580602	−2.7621872	0.00574155	0.08279327	Up
ENSG00000226562	2.83969294	−4.8	2.15712697	−2.2323878	0.02558934	0.19291473	Up
ENSG00000267653	18.6393025	−4.6	1.18355532	−3.8857992	0.00010199	0.00535485	Up
ENSG00000298690	2.40410301	−4.6	1.31131045	−3.4816176	0.0004984	0.01688615	Up
ENSG00000304163	3.60829109	−4.6	1.63774551	−2.8282687	0.00468005	0.07329761	Up
ENSG00000300437	2.48886314	−4.6	1.7087183	−2.7103252	0.00672173	0.09071955	Up
ENSG00000293757	2.45471648	−4.6	2.2150069	−2.0801695	0.03750999	0.23607729	Up
ENSG00000285649	2.24907601	−4.5	1.49009445	−3.006602	0.00264185	NA	Up
ENSG00000276831	2.26891435	−4.5	2.15967333	−2.084345	0.03712879	NA	Up
ENSG00000224239	3.14919595	−4.4	1.21015928	−3.6694311	0.00024309	0.00996896	Up
ENSG00000296719	2.07942702	−4.4	1.29583942	−3.3770964	0.00073255	NA	Up
ENSG00000295613	2.11341551	−4.4	1.77626226	−2.4722183	0.01342775	NA	Up
ENSG00000298074	43.2261772	−4.3	0.68673232	−6.227569	4.74 × 10^−10^	3.71 × 10^−7^	Up
ENSG00000274478	3.94265842	−4.3	1.38362988	−3.0753408	0.00210262	0.04461646	Up
ENSG00000227487	4.06682931	−4.3	1.58140449	−2.721663	0.00649543	0.08932244	Up
ENSG00000231132	1.92513856	−4.3	1.86805998	−2.2793749	0.02264479	NA	Up
ENSG00000296003	1.94909901	−4.3	1.89990295	−2.2485052	0.02454399	NA	Up
ENSG00000298193	1.92079493	−4.3	1.96128058	−2.16822	0.03014195	NA	Up
ENSG00000285936	1.97756865	−4.3	2.1797068	−1.9693765	0.04890988	NA	Up
ENSG00000300255	1.91034707	−4.2	1.34504573	−3.1580307	0.00158839	NA	Up
ENSG00000286468	1.92446954	−4.2	1.69334955	−2.5096469	0.0120852	NA	Up
ENSG00000297456	1.79753544	−4.2	1.97057075	−2.1102525	0.03483661	NA	Up
ENSG00000264007	2.74415058	−4.2	2.02575856	−2.0950347	0.0361679	0.23135695	Up
ENSG00000231419	10.5586343	−4.1	0.95511704	−4.2721184	1.94 × 10^−5^	0.00153673	Up
ENSG00000297199	9.18829618	−4.1	1.06625967	−3.8898188	0.00010032	0.00528289	Up
ENSG00000305011	2.48231308	−4.1	1.18927314	−3.4505029	0.00055954	0.01805428	Up
ENSG00000229495	1.74392169	−4.1	1.5299145	−2.6866744	0.00721673	NA	Up
ENSG00000275358	3.47818747	−4.1	1.70141215	−2.4009139	0.01635418	0.15200984	Up
ENSG00000300949	1.67487121	−4.1	1.84983805	−2.1963248	0.0280687	NA	Up
ENSG00000286411	2.505807	−4.1	1.89232231	−2.1742414	0.02968701	0.20751407	Up
ENSG00000285686	3.5641727	−4.1	1.91437549	−2.1534663	0.03128205	0.21402918	Up
ENSG00000286818	6.03882055	−4	1.25554434	−3.2155005	0.00130217	0.0325075	Up
ENSG00000294628	1.600431	−4	1.54122597	−2.5909005	0.00957252	NA	Up
ENSG00000300397	3.36778179	−4	1.71935962	−2.3439341	0.01908155	0.1658802	Up
ENSG00000234944	3.24048221	−4	1.72676428	−2.2928222	0.02185824	0.1789809	Up
ENSG00000204971	3.44993353	5.4	1.39793787	3.84619167	0.00011997	0.00603911	Down
ENSG00000298268	3.46510039	5.4	1.48640556	3.60463808	0.00031259	0.01198425	Down
ENSG00000258183	4.83347254	5.4	1.56660674	3.41522649	0.00063729	0.01970541	Down
ENSG00000303389	67.4491212	5.4	1.59960987	3.39016959	0.00069849	0.02101712	Down
ENSG00000249667	3.57232375	5.4	1.78879945	3.02570312	0.00248056	0.04947514	Down
ENSG00000249993	3.51827941	5.4	1.81714131	2.96381834	0.00303848	0.05650863	Down
ENSG00000310062	3.48578139	5.4	1.8301654	2.93439046	0.00334204	0.06010549	Down
ENSG00000304111	3.54345937	5.4	1.85545923	2.90663633	0.00365338	0.06364457	Down
ENSG00000308399	3.52372208	5.4	2.06650839	2.60844294	0.00909552	0.10839416	Down
ENSG00000290670	3.52399192	5.4	2.08902577	2.57982723	0.00988498	0.1137742	Down
ENSG00000300640	3.53914029	5.4	2.12685373	2.53647022	0.01119763	0.12259215	Down
ENSG00000306164	3.45792879	5.4	2.15200616	2.49173312	0.01271215	0.13155437	Down
ENSG00000250822	3.60090773	5.4	2.48073063	2.18592409	0.02882116	0.20492453	Down
ENSG00000290840	5.17888445	5.5	1.50803576	3.61858637	0.00029622	0.01151552	Down
ENSG00000300947	3.78576114	5.5	1.63901592	3.34744641	0.0008156	0.02356484	Down
ENSG00000289707	3.82574307	5.5	1.73352772	3.18330533	0.00145604	0.03503209	Down
ENSG00000300565	3.73979054	5.5	1.7681675	3.09858123	0.0019445	0.04225892	Down
ENSG00000250348	3.81618728	5.5	2.45002286	2.2518587	0.0243312	0.1891298	Down
ENSG00000230838	194.501423	5.6	0.70577156	7.98793817	1.37 × 10^−15^	3.81 × 10^−12^	Down
ENSG00000298768	22.8322869	5.6	0.95737791	5.85599323	4.74 × 10^−9^	2.68 × 10^−6^	Down
ENSG00000224099	4.19313483	5.6	1.77249083	3.18113846	0.00146698	0.03521975	Down
ENSG00000308513	5.8501961	5.6	1.88810388	2.97828392	0.00289867	0.05482292	Down
ENSG00000295056	4.32612076	5.7	1.36349558	4.18252197	2.88 × 10^−5^	0.00207142	Down
ENSG00000259937	8.83425397	5.7	1.44977142	3.95282857	7.72 × 10^−5^	0.00441933	Down
ENSG00000288015	4.27678936	5.7	1.46219699	3.87282695	0.00010758	0.00555274	Down
ENSG00000308731	4.33753956	5.7	1.55232652	3.66561513	0.00024675	0.01005271	Down
ENSG00000288049	4.24690662	5.7	1.69421782	3.34957101	0.00080937	0.02348628	Down
ENSG00000253554	4.33106158	5.7	1.7507366	3.24846626	0.00116029	0.03006132	Down
ENSG00000232596	6.40357577	5.8	1.51402082	3.79913474	0.0001452	0.00686811	Down
ENSG00000298049	4.64546188	5.8	1.77170343	3.26603666	0.00109064	0.02881119	Down
ENSG00000294222	17.6914436	5.9	1.47771016	4.01898815	5.84 × 10^−5^	0.00358626	Down
ENSG00000293442	155.369799	5.9	1.48639466	3.93570269	8.30 × 10^−5^	0.00466193	Down
ENSG00000264985	5.19724286	5.9	1.56786443	3.79214101	0.00014935	0.00698876	Down
ENSG00000294508	5.07296798	5.9	1.75683438	3.36730502	0.00075907	0.02236649	Down
ENSG00000251670	5.27532038	6	1.25670454	4.7454957	2.08 × 10^−6^	0.00029154	Down
ENSG00000233854	5.74368619	6.1	1.30938943	4.65113408	3.30 × 10^−6^	0.0004047	Down
ENSG00000286028	5.90442962	6.1	1.45659643	4.21037416	2.55 × 10^−5^	0.00189082	Down
ENSG00000298839	32.2989896	6.2	1.03885417	5.94096616	2.83 × 10^−9^	1.80 × 10^−6^	Down
ENSG00000293047	12.2817787	6.2	1.11489762	5.57238667	2.51 × 10^−8^	8.72 × 10^−6^	Down
ENSG00000249343	8.40798677	6.2	1.55287593	3.96157443	7.45 × 10^−5^	0.00429032	Down
ENSG00000176728	57.9891864	6.5	1.11309151	5.79756785	6.73 × 10^−9^	3.12 × 10^−6^	Down
ENSG00000271216	15.2594445	6.5	1.36837552	4.77529373	1.79 × 10^−6^	0.0002611	Down
ENSG00000229236	10.3349528	6.5	1.35769107	4.75996551	1.94 × 10^−6^	0.00027647	Down
ENSG00000266604	8.70411474	6.7	1.56681229	4.27280749	1.93 × 10^−5^	0.00153597	Down
ENSG00000231131	18.9261187	6.8	1.05759833	6.47436337	9.52 × 10^−11^	8.31 × 10^−8^	Down
ENSG00000249306	151.620051	6.9	0.82875406	8.3133538	9.30 × 10^−17^	4.06 × 10^−13^	Down
ENSG00000260197	19.7348345	6.9	1.32944921	5.20130859	1.98 × 10^−7^	4.72 × 10^−5^	Down
ENSG00000229876	10.5213785	7	1.42708418	4.88236894	1.05 × 10^−6^	0.00017407	Down
ENSG00000231535	27.0797506	7.4	1.32158065	5.58007893	2.40 × 10^−8^	8.44 × 10^−6^	Down
ENSG00000291033	276.125148	7.7	1.03227535	7.48581379	7.11 × 10^−14^	1.21 × 10^−10^	Down

## Data Availability

The datasets analyzed during the current study are available in the NCBI database, accession numbers SRX10431608–SRX10431613 and SRX10431614–SRX10431619.

## Supplementary Materials

The following supporting information can be downloaded at: https://www.mdpi.com/article/10.3390/ijms26168058/s1.

## Abbreviations

DDH	Developmental dysplasia of the hip
LncRNA	Long non-coding RNA
GO	Gene Ontology
KEGG	Kyoto Encyclopedia of Genes and Genomes
DEGs	Differentially expressed genes
MF	Molecular function
BP	Biological process
CC	Cellular component
MMP13	Matrix Metalloproteinase 13
MMP3	Matrix Metalloproteinase 3
GDF5	Growth Differentiation Factor 5
DKK1	Dickkopf WNT Signaling Pathway Inhibitor 1
WIF1	Wnt Inhibitor Factor 1
MYOC	Myocilin
IRX	Iroquois Homeobox
